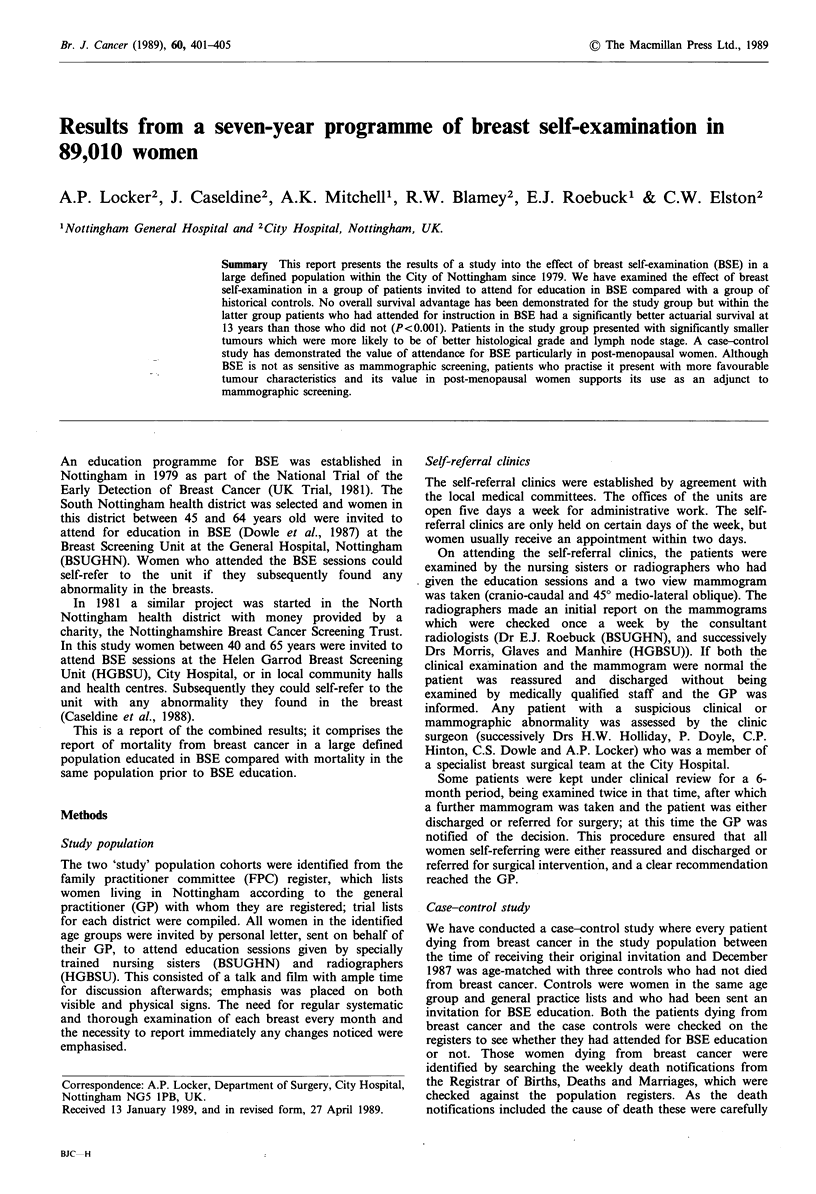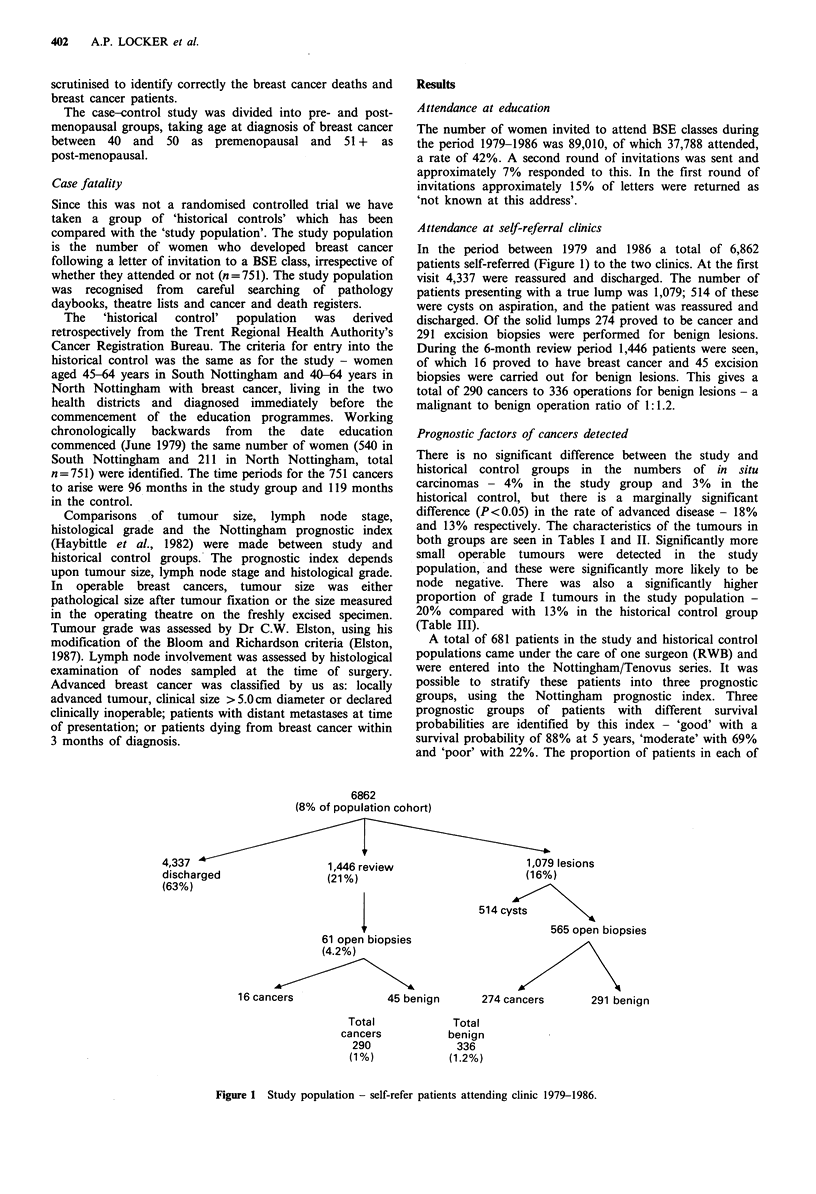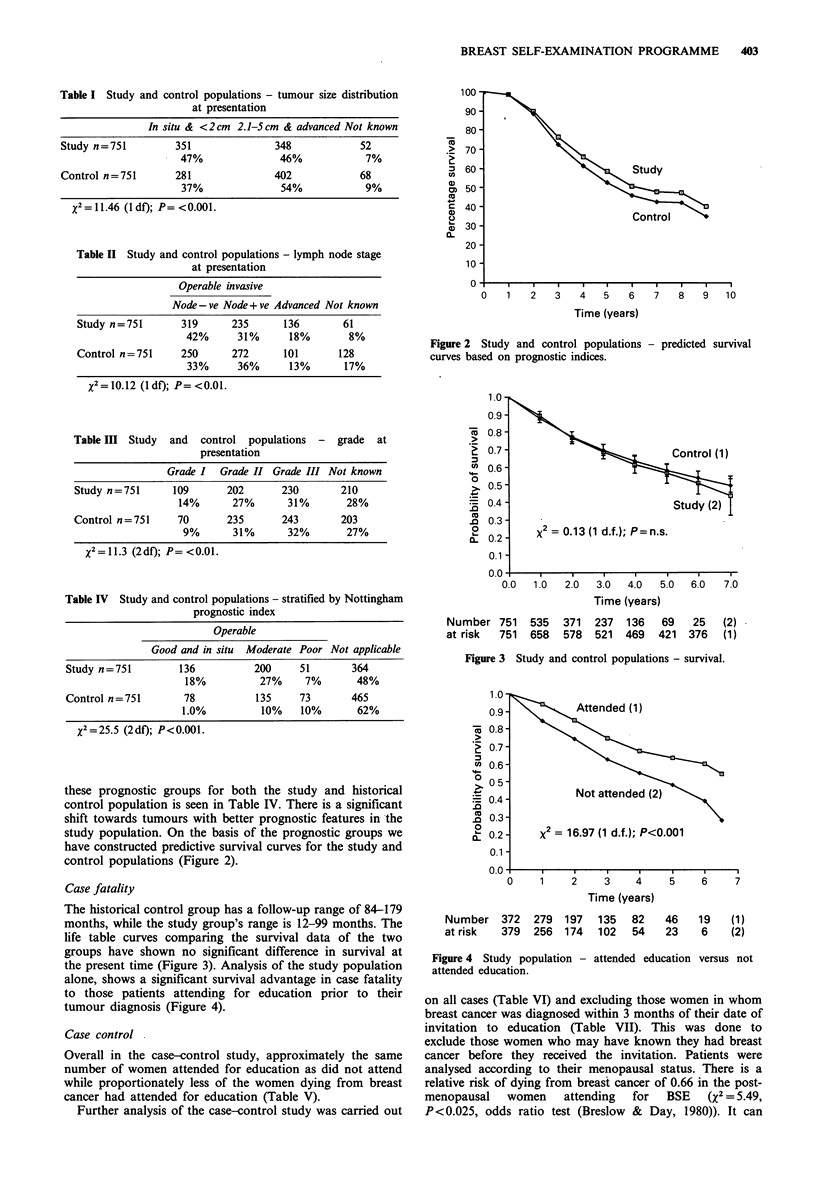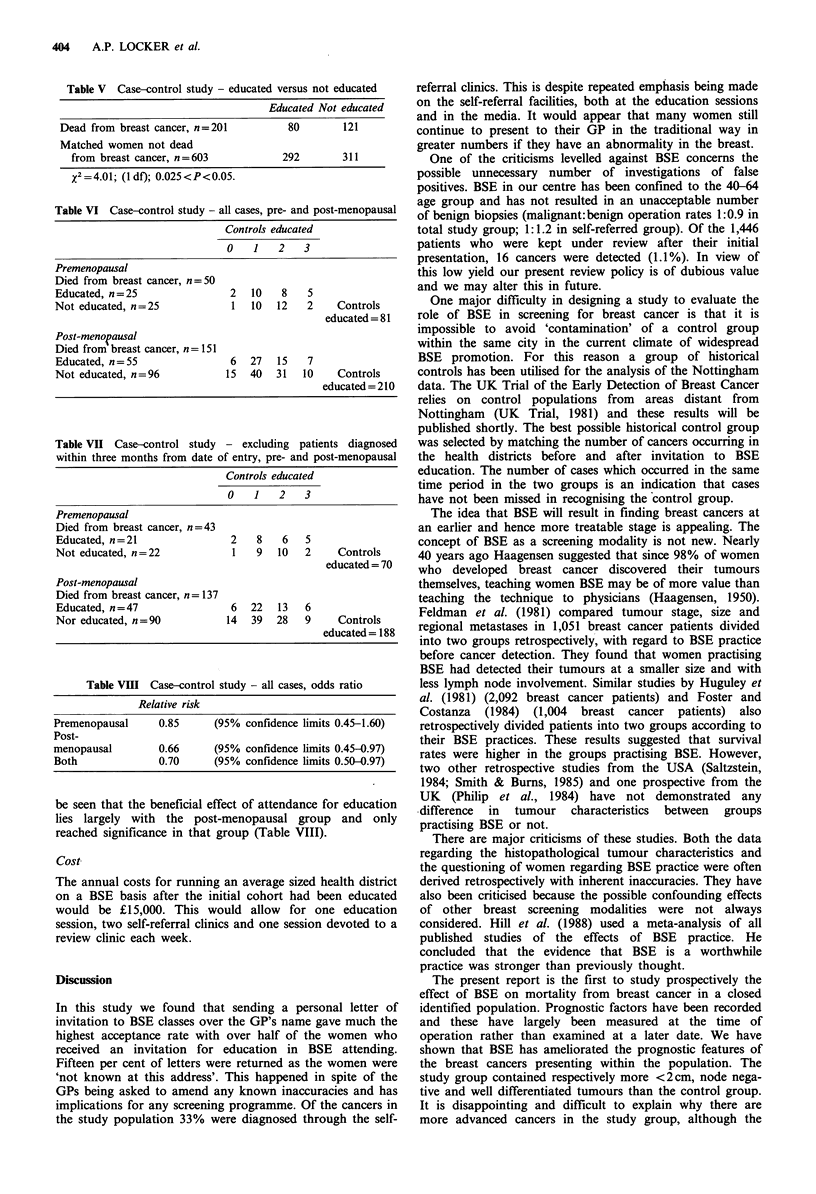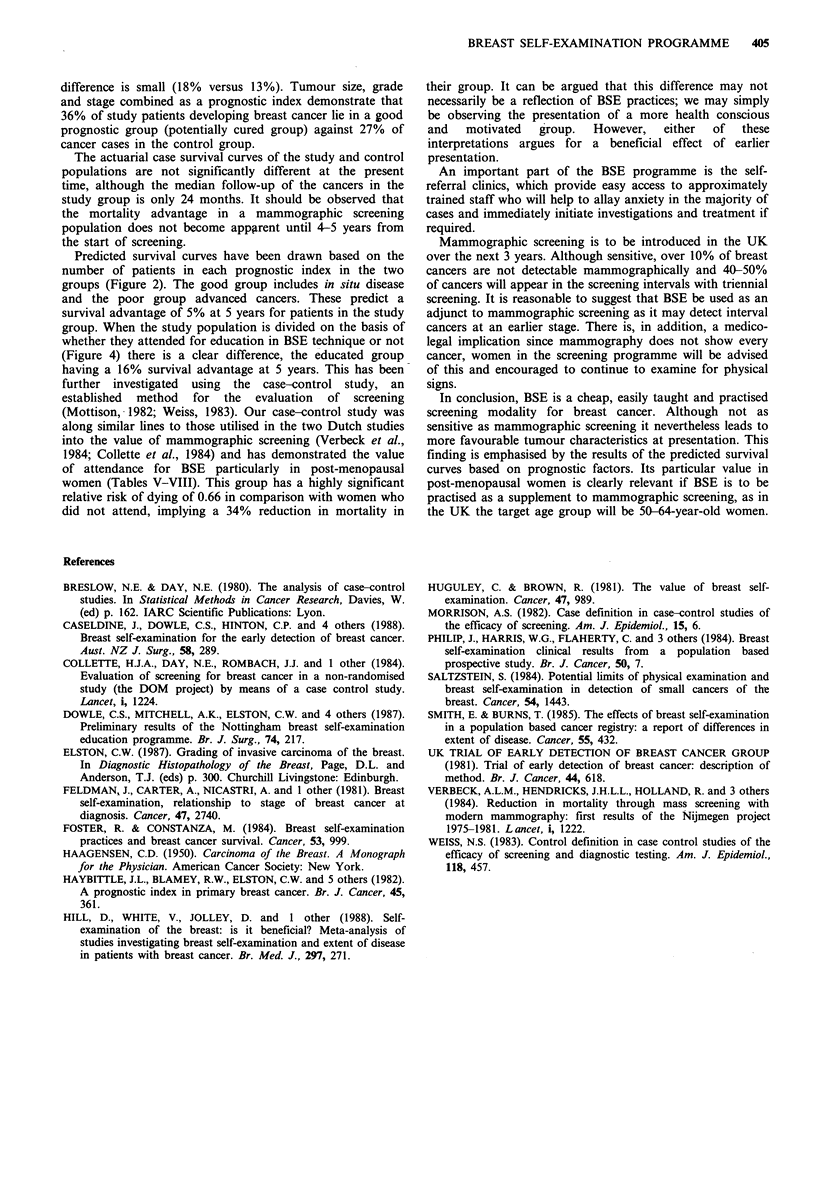# Results from a seven-year programme of breast self-examination in 89,010 women.

**DOI:** 10.1038/bjc.1989.294

**Published:** 1989-09

**Authors:** A. P. Locker, J. Caseldine, A. K. Mitchell, R. W. Blamey, E. J. Roebuck, C. W. Elston

**Affiliations:** Nottingham General Hospital, UK.

## Abstract

This report presents the results of a study into the effect of breast self-examination (BSE) in a large defined population within the City of Nottingham since 1979. We have examined the effect of breast self-examination in a group of patients invited to attend for education in BSE compared with a group of historical controls. No overall survival advantage has been demonstrated for the study group but within the latter group patients who had attended for instruction in BSE had a significantly better actuarial survival at 13 years than those who did not (P less than 0.001). Patients in the study group presented with significantly smaller tumours which were more likely to be of better histological grade and lymph node stage. A case-control study has demonstrated the value of attendance for BSE particularly in post-menopausal women. Although BSE is not as sensitive as mammographic screening, patients who practise it present with more favourable tumour characteristics and its value in post-menopausal women supports its use as an adjunct to mammographic screening.


*Br. J. Cancer* (1989), **60**, 401–405 © The Macmillan Press Ltd., 1989

### Results from a seven-year programme of breast self-examination in 89,010 women

A.P. Locker[2], J. Caseldine[2], A.K. Mitchell[1], R.W. Blamey[2], E.J. Roebuck[1] & C.W. Elston[2]

[1]*Nottingham General Hospital and* [2]*City Hospital, Nottingham, UK.*

**Summary** This report presents the results of a study into the effect of breast self-examination (BSE) in a large defined population within the City of Nottingham since 1979. We have examined the effect of breast self-examination in a group of patients invited to attend for education in BSE compared with a group of historical controls. No overall survival advantage has been demonstrated for the study group but within the latter group patients who had attended for instruction in BSE had a significantly better actuarial survival at 13 years than those who did not ($P<0.001$). Patients in the study group presented with significantly smaller tumours which were more likely to be of better histological grade and lymph node stage. A case–control study has demonstrated the value of attendance for BSE particularly in post-menopausal women. Although BSE is not as sensitive as mammographic screening, patients who practise it present with more favourable tumour characteristics and its value in post-menopausal women supports its use as an adjunct to mammographic screening.

An education programme for BSE was established in Nottingham in 1979 as part of the National Trial of the Early Detection of Breast Cancer (UK Trial, 1981). The South Nottingham health district was selected and women in this district between 45 and 64 years old were invited to attend for education in BSE (Dowle *et al.*, 1987) at the Breast Screening Unit at the General Hospital, Nottingham (BSUGHN). Women who attended the BSE sessions could self-refer to the unit if they subsequently found any abnormality in the breasts.

In 1981 a similar project was started in the North Nottingham health district with money provided by a charity, the Nottinghamshire Breast Cancer Screening Trust. In this study women between 40 and 65 years were invited to attend BSE sessions at the Helen Garrod Breast Screening Unit (HGBSU), City Hospital, or in local community halls and health centres. Subsequently they could self-refer to the unit with any abnormality they found in the breast (Caseldine *et al.*, 1988).

This is a report of the combined results; it comprises the report of mortality from breast cancer in a large defined population educated in BSE compared with mortality in the same population prior to BSE education.

### Methods

### *Study population*

The two 'study' population cohorts were identified from the family practitioner committee (FPC) register, which lists women living in Nottingham according to the general practitioner (GP) with whom they are registered; trial lists for each district were compiled. All women in the identified age groups were invited by personal letter, sent on behalf of their GP, to attend education sessions given by specially trained nursing sisters (BSUGHN) and radiographers (HGBSU). This consisted of a talk and film with ample time for discussion afterwards; emphasis was placed on both visible and physical signs. The need for regular systematic and thorough examination of each breast every month and the necessity to report immediately any changes noticed were emphasised.

Correspondence: A.P. Locker, Department of Surgery, City Hospital, Nottingham NG5 1PB, UK.
Received 13 January 1989, and in revised form, 27 April 1989.

### *Self-referral clinics*

The self-referral clinics were established by agreement with the local medical committees. The offices of the units are open five days a week for administrative work. The self-referral clinics are only held on certain days of the week, but women usually receive an appointment within two days.

On attending the self-referral clinics, the patients were examined by the nursing sisters or radiographers who had given the education sessions and a two view mammogram was taken (cranio-caudal and 45° medio-lateral oblique). The radiographers made an initial report on the mammograms which were checked once a week by the consultant radiologists (Dr E.J. Roebuck (BSUGHN), and successively Drs Morris, Glaves and Manhire (HGBSU)). If both the clinical examination and the mammogram were normal the patient was reassured and discharged without being examined by medically qualified staff and the GP was informed. Any patient with a suspicious clinical or mammographic abnormality was assessed by the clinic surgeon (successively Drs H.W. Holliday, P. Doyle, C.P. Hinton, C.S. Dowle and A.P. Locker) who was a member of a specialist breast surgical team at the City Hospital.

Some patients were kept under clinical review for a 6-month period, being examined twice in that time, after which a further mammogram was taken and the patient was either discharged or referred for surgery; at this time the GP was notified of the decision. This procedure ensured that all women self-referring were either reassured and discharged or referred for surgical intervention, and a clear recommendation reached the GP.

### *Case–control study*

We have conducted a case–control study where every patient dying from breast cancer in the study population between the time of receiving their original invitation and December 1987 was age-matched with three controls who had not died from breast cancer. Controls were women in the same age group and general practice lists and who had been sent an invitation for BSE education. Both the patients dying from breast cancer and the case controls were checked on the registers to see whether they had attended for BSE education or not. Those women dying from breast cancer were identified by searching the weekly death notifications from the Registrar of Births, Deaths and Marriages, which were checked against the population registers. As the death notifications included the cause of death these were carefully scrutinised to identify correctly the breast cancer deaths and breast cancer patients.

The case–control study was divided into pre- and post-menopausal groups, taking age at diagnosis of breast cancer between 40 and 50 as premenopausal and 51+ as post-menopausal.

*Case fatality*

Since this was not a randomised controlled trial we have taken a group of 'historical controls' which has been compared with the 'study population'. The study population is the number of women who developed breast cancer following a letter of invitation to a BSE class, irrespective of whether they attended or not ($n=751$). The study population was recognised from careful searching of pathology daybooks, theatre lists and cancer and death registers.

The 'historical control' population was derived retrospectively from the Trent Regional Health Authority's Cancer Registration Bureau. The criteria for entry into the historical control was the same as for the study – women aged 45–64 years in South Nottingham and 40–64 years in North Nottingham with breast cancer, living in the two health districts and diagnosed immediately before the commencement of the education programmes. Working chronologically backwards from the date education commenced (June 1979) the same number of women (540 in South Nottingham and 211 in North Nottingham, total $n=751$) were identified. The time periods for the 751 cancers to arise were 96 months in the study group and 119 months in the control.

Comparisons of tumour size, lymph node stage, histological grade and the Nottingham prognostic index (Haybittle *et al.*, 1982) were made between study and historical control groups. The prognostic index depends upon tumour size, lymph node stage and histological grade. In operable breast cancers, tumour size was either pathological size after tumour fixation or the size measured in the operating theatre on the freshly excised specimen. Tumour grade was assessed by Dr C.W. Elston, using his modification of the Bloom and Richardson criteria (Elston, 1987). Lymph node involvement was assessed by histological examination of nodes sampled at the time of surgery. Advanced breast cancer was classified by us as: locally advanced tumour, clinical size >5.0 cm diameter or declared clinically inoperable; patients with distant metastases at time of presentation; or patients dying from breast cancer within 3 months of diagnosis.

### Results

*Attendance at education*

The number of women invited to attend BSE classes during the period 1979–1986 was 89,010, of which 37,788 attended, a rate of 42%. A second round of invitations was sent and approximately 7% responded to this. In the first round of invitations approximately 15% of letters were returned as 'not known at this address'.

*Attendance at self-referral clinics*

In the period between 1979 and 1986 a total of 6,862 patients self-referred (Figure 1) to the two clinics. At the first visit 4,337 were reassured and discharged. The number of patients presenting with a true lump was 1,079; 514 of these were cysts on aspiration, and the patient was reassured and discharged. Of the solid lumps 274 proved to be cancer and 291 excision biopsies were performed for benign lesions. During the 6-month review period 1,446 patients were seen, of which 16 proved to have breast cancer and 45 excision biopsies were carried out for benign lesions. This gives a total of 290 cancers to 336 operations for benign lesions – a malignant to benign operation ratio of 1:1.2.

*Prognostic factors of cancers detected*

There is no significant difference between the study and historical control groups in the numbers of *in situ* carcinomas – 4% in the study group and 3% in the historical control, but there is a marginally significant difference ($P<0.05$) in the rate of advanced disease – 18% and 13% respectively. The characteristics of the tumours in both groups are seen in Tables I and II. Significantly more small operable tumours were detected in the study population, and these were significantly more likely to be node negative. There was also a significantly higher proportion of grade I tumours in the study population – 20% compared with 13% in the historical control group (Table III).

A total of 681 patients in the study and historical control populations came under the care of one surgeon (RWB) and were entered into the Nottingham/Tenovus series. It was possible to stratify these patients into three prognostic groups, using the Nottingham prognostic index. Three prognostic groups of patients with different survival probabilities are identified by this index – 'good' with a survival probability of 88% at 5 years, 'moderate' with 69% and 'poor' with 22%. The proportion of patients in each of

**Figure 1** Study population – self-refer patients attending clinic 1979–1986.

**Table I** Study and control populations – tumour size distribution at presentation

| | *In situ & <2 cm* | *2.1–5 cm & advanced* | *Not known* |
|---|---|---|---|
| Study $n=751$ | 351<br>47% | 348<br>46% | 52<br>7% |
| Control $n=751$ | 281<br>37% | 402<br>54% | 68<br>9% |

$\chi^2=11.46$ (1 df); $P=<0.001$.

**Table II** Study and control populations – lymph node stage at presentation

| | *Operable invasive* | | | |
|---|---|---|---|---|
| | *Node – ve* | *Node + ve* | *Advanced* | *Not known* |
| Study $n=751$ | 319<br>42% | 235<br>31% | 136<br>18% | 61<br>8% |
| Control $n=751$ | 250<br>33% | 272<br>36% | 101<br>13% | 128<br>17% |

$\chi^2=10.12$ (1 df); $P=<0.01$.

**Table III** Study and control populations – grade at presentation

| | *Grade I* | *Grade II* | *Grade III* | *Not known* |
|---|---|---|---|---|
| Study $n=751$ | 109<br>14% | 202<br>27% | 230<br>31% | 210<br>28% |
| Control $n=751$ | 70<br>9% | 235<br>31% | 243<br>32% | 203<br>27% |

$\chi^2=11.3$ (2 df); $P=<0.01$.

**Table IV** Study and control populations – stratified by Nottingham prognostic index

| | *Operable* | | | |
|---|---|---|---|---|
| | *Good and in situ* | *Moderate* | *Poor* | *Not applicable* |
| Study $n=751$ | 136<br>18% | 200<br>27% | 51<br>7% | 364<br>48% |
| Control $n=751$ | 78<br>1.0% | 135<br>10% | 73<br>10% | 465<br>62% |

$\chi^2=25.5$ (2 df); $P<0.001$.

these prognostic groups for both the study and historical control population is seen in Table IV. There is a significant shift towards tumours with better prognostic features in the study population. On the basis of the prognostic groups we have constructed predictive survival curves for the study and control populations (Figure 2).

### *Case fatality*

The historical control group has a follow-up range of 84–179 months, while the study group's range is 12–99 months. The life table curves comparing the survival data of the two groups have shown no significant difference in survival at the present time (Figure 3). Analysis of the study population alone, shows a significant survival advantage in case fatality to those patients attending for education prior to their tumour diagnosis (Figure 4).

### *Case control*

Overall in the case–control study, approximately the same number of women attended for education as did not attend while proportionately less of the women dying from breast cancer had attended for education (Table V).

Further analysis of the case–control study was carried out

**Figure 2** Study and control populations – predicted survival curves based on prognostic indices.

**Figure 3** Study and control populations – survival.

**Figure 4** Study population – attended education versus not attended education.

on all cases (Table VI) and excluding those women in whom breast cancer was diagnosed within 3 months of their date of invitation to education (Table VII). This was done to exclude those women who may have known they had breast cancer before they received the invitation. Patients were analysed according to their menopausal status. There is a relative risk of dying from breast cancer of 0.66 in the post-menopausal women attending for BSE ($\chi^2=5.49$, $P<0.025$, odds ratio test (Breslow & Day, 1980)). It can

**Table V** Case–control study – educated versus not educated

| | *Educated* | *Not educated* |
|---|---|---|
| Dead from breast cancer, $n=201$ | 80 | 121 |
| Matched women not dead from breast cancer, $n=603$ | 292 | 311 |

$\chi^2=4.01$; (1 df); $0.025<P<0.05$.

**Table VI** Case–control study – all cases, pre- and post-menopausal

| | *Controls educated* | | | | |
|---|---|---|---|---|---|
| | *0* | *1* | *2* | *3* | |
| *Premenopausal* | | | | | |
| Died from breast cancer, $n=50$ | | | | | |
| Educated, $n=25$ | 2 | 10 | 8 | 5 | |
| Not educated, $n=25$ | 1 | 10 | 12 | 2 | Controls educated = 81 |
| *Post-menopausal* | | | | | |
| Died from breast cancer, $n=151$ | | | | | |
| Educated, $n=55$ | 6 | 27 | 15 | 7 | |
| Not educated, $n=96$ | 15 | 40 | 31 | 10 | Controls educated = 210 |

**Table VII** Case–control study – excluding patients diagnosed within three months from date of entry, pre- and post-menopausal

| | *Controls educated* | | | | |
|---|---|---|---|---|---|
| | *0* | *1* | *2* | *3* | |
| *Premenopausal* | | | | | |
| Died from breast cancer, $n=43$ | | | | | |
| Educated, $n=21$ | 2 | 8 | 6 | 5 | |
| Not educated, $n=22$ | 1 | 9 | 10 | 2 | Controls educated = 70 |
| *Post-menopausal* | | | | | |
| Died from breast cancer, $n=137$ | | | | | |
| Educated, $n=47$ | 6 | 22 | 13 | 6 | |
| Nor educated, $n=90$ | 14 | 39 | 28 | 9 | Controls educated = 188 |

**Table VIII** Case–control study – all cases, odds ratio

| | *Relative risk* | |
|---|---|---|
| Premenopausal | 0.85 | (95% confidence limits 0.45–1.60) |
| Post-menopausal | 0.66 | (95% confidence limits 0.45–0.97) |
| Both | 0.70 | (95% confidence limits 0.50–0.97) |

be seen that the beneficial effect of attendance for education lies largely with the post-menopausal group and only reached significance in that group (Table VIII).

*Cost*

The annual costs for running an average sized health district on a BSE basis after the initial cohort had been educated would be £15,000. This would allow for one education session, two self-referral clinics and one session devoted to a review clinic each week.

### Discussion

In this study we found that sending a personal letter of invitation to BSE classes over the GP's name gave much the highest acceptance rate with over half of the women who received an invitation for education in BSE attending. Fifteen per cent of letters were returned as the women were 'not known at this address'. This happened in spite of the GPs being asked to amend any known inaccuracies and has implications for any screening programme. Of the cancers in the study population 33% were diagnosed through the self-referral clinics. This is despite repeated emphasis being made on the self-referral facilities, both at the education sessions and in the media. It would appear that many women still continue to present to their GP in the traditional way in greater numbers if they have an abnormality in the breast.

One of the criticisms levelled against BSE concerns the possible unnecessary number of investigations of false positives. BSE in our centre has been confined to the 40–64 age group and has not resulted in an unacceptable number of benign biopsies (malignant:benign operation rates 1:0.9 in total study group; 1:1.2 in self-referred group). Of the 1,446 patients who were kept under review after their initial presentation, 16 cancers were detected (1.1%). In view of this low yield our present review policy is of dubious value and we may alter this in future.

One major difficulty in designing a study to evaluate the role of BSE in screening for breast cancer is that it is impossible to avoid 'contamination' of a control group within the same city in the current climate of widespread BSE promotion. For this reason a group of historical controls has been utilised for the analysis of the Nottingham data. The UK Trial of the Early Detection of Breast Cancer relies on control populations from areas distant from Nottingham (UK Trial, 1981) and these results will be published shortly. The best possible historical control group was selected by matching the number of cancers occurring in the health districts before and after invitation to BSE education. The number of cases which occurred in the same time period in the two groups is an indication that cases have not been missed in recognising the control group.

The idea that BSE will result in finding breast cancers at an earlier and hence more treatable stage is appealing. The concept of BSE as a screening modality is not new. Nearly 40 years ago Haagensen suggested that since 98% of women who developed breast cancer discovered their tumours themselves, teaching women BSE may be of more value than teaching the technique to physicians (Haagensen, 1950). Feldman *et al.* (1981) compared tumour stage, size and regional metastases in 1,051 breast cancer patients divided into two groups retrospectively, with regard to BSE practice before cancer detection. They found that women practising BSE had detected their tumours at a smaller size and with less lymph node involvement. Similar studies by Huguley *et al.* (1981) (2,092 breast cancer patients) and Foster and Costanza (1984) (1,004 breast cancer patients) also retrospectively divided patients into two groups according to their BSE practices. These results suggested that survival rates were higher in the groups practising BSE. However, two other retrospective studies from the USA (Saltzstein, 1984; Smith & Burns, 1985) and one prospective from the UK (Philip *et al.*, 1984) have not demonstrated any difference in tumour characteristics between groups practising BSE or not.

There are major criticisms of these studies. Both the data regarding the histopathological tumour characteristics and the questioning of women regarding BSE practice were often derived retrospectively with inherent inaccuracies. They have also been criticised because the possible confounding effects of other breast screening modalities were not always considered. Hill *et al.* (1988) used a meta-analysis of all published studies of the effects of BSE practice. He concluded that the evidence that BSE is a worthwhile practice was stronger than previously thought.

The present report is the first to study prospectively the effect of BSE on mortality from breast cancer in a closed identified population. Prognostic factors have been recorded and these have largely been measured at the time of operation rather than examined at a later date. We have shown that BSE has ameliorated the prognostic features of the breast cancers presenting within the population. The study group contained respectively more <2 cm, node negative and well differentiated tumours than the control group. It is disappointing and difficult to explain why there are more advanced cancers in the study group, although the difference is small (18% versus 13%). Tumour size, grade and stage combined as a prognostic index demonstrate that 36% of study patients developing breast cancer lie in a good prognostic group (potentially cured group) against 27% of cancer cases in the control group.

The actuarial case survival curves of the study and control populations are not significantly different at the present time, although the median follow-up of the cancers in the study group is only 24 months. It should be observed that the mortality advantage in a mammographic screening population does not become apparent until 4–5 years from the start of screening.

Predicted survival curves have been drawn based on the number of patients in each prognostic index in the two groups (Figure 2). The good group includes *in situ* disease and the poor group advanced cancers. These predict a survival advantage of 5% at 5 years for patients in the study group. When the study population is divided on the basis of whether they attended for education in BSE technique or not (Figure 4) there is a clear difference, the educated group having a 16% survival advantage at 5 years. This has been further investigated using the case–control study, an established method for the evaluation of screening (Mottison, 1982; Weiss, 1983). Our case–control study was along similar lines to those utilised in the two Dutch studies into the value of mammographic screening (Verbeck *et al.*, 1984; Collette *et al.*, 1984) and has demonstrated the value of attendance for BSE particularly in post-menopausal women (Tables V–VIII). This group has a highly significant relative risk of dying of 0.66 in comparison with women who did not attend, implying a 34% reduction in mortality in their group. It can be argued that this difference may not necessarily be a reflection of BSE practices; we may simply be observing the presentation of a more health conscious and motivated group. However, either of these interpretations argues for a beneficial effect of earlier presentation.

An important part of the BSE programme is the self-referral clinics, which provide easy access to approximately trained staff who will help to allay anxiety in the majority of cases and immediately initiate investigations and treatment if required.

Mammographic screening is to be introduced in the UK over the next 3 years. Although sensitive, over 10% of breast cancers are not detectable mammographically and 40–50% of cancers will appear in the screening intervals with triennial screening. It is reasonable to suggest that BSE be used as an adjunct to mammographic screening as it may detect interval cancers at an earlier stage. There is, in addition, a medico-legal implication since mammography does not show every cancer, women in the screening programme will be advised of this and encouraged to continue to examine for physical signs.

In conclusion, BSE is a cheap, easily taught and practised screening modality for breast cancer. Although not as sensitive as mammographic screening it nevertheless leads to more favourable tumour characteristics at presentation. This finding is emphasised by the results of the predicted survival curves based on prognostic factors. Its particular value in post-menopausal women is clearly relevant if BSE is to be practised as a supplement to mammographic screening, as in the UK the target age group will be 50–64-year-old women.

### References

BRESLOW, N.E. & DAY, N.E. (1980). The analysis of case–control studies. In *Statistical Methods in Cancer Research*, Davies, W. (ed) p. 162. IARC Scientific Publications: Lyon.

CASELDINE, J., DOWLE, C.S., HINTON, C.P. and 4 others (1988). Breast self-examination for the early detection of breast cancer. *Aust. NZ J. Surg.*, **58**, 289.

COLLETTE, H.J.A., DAY, N.E., ROMBACH, J.J. and 1 other (1984). Evaluation of screening for breast cancer in a non-randomised study (the DOM project) by means of a case control study. *Lancet*, **i**, 1224.

DOWLE, C.S., MITCHELL, A.K., ELSTON, C.W. and 4 others (1987). Preliminary results of the Nottingham breast self-examination education programme. *Br. J. Surg.*, **74**, 217.

ELSTON, C.W. (1987). Grading of invasive carcinoma of the breast. In *Diagnostic Histopathology of the Breast*, Page, D.L. and Anderson, T.J. (eds) p. 300. Churchill Livingstone: Edinburgh.

FELDMAN, J., CARTER, A., NICASTRI, A. and 1 other (1981). Breast self-examination, relationship to stage of breast cancer at diagnosis. *Cancer*, **47**, 2740.

FOSTER, R. & CONSTANZA, M. (1984). Breast self-examination practices and breast cancer survival. *Cancer*, **53**, 999.

HAAGENSEN, C.D. (1950). *Carcinoma of the Breast. A Monograph for the Physician*. American Cancer Society: New York.

HAYBITTLE, J.L., BLAMEY, R.W., ELSTON, C.W. and 5 others (1982). A prognostic index in primary breast cancer. *Br. J. Cancer*, **45**, 361.

HILL, D., WHITE, V., JOLLEY, D. and 1 other (1988). Self-examination of the breast: is it beneficial? Meta-analysis of studies investigating breast self-examination and extent of disease in patients with breast cancer. *Br. Med. J.*, **297**, 271.

HUGULEY, C. & BROWN, R. (1981). The value of breast self-examination. *Cancer*, **47**, 989.

MORRISON, A.S. (1982). Case definition in case–control studies of the efficacy of screening. *Am. J. Epidemiol.*, **15**, 6.

PHILIP, J., HARRIS, W.G., FLAHERTY, C. and 3 others (1984). Breast self-examination clinical results from a population based prospective study. *Br. J. Cancer*, **50**, 7.

SALTZSTEIN, S. (1984). Potential limits of physical examination and breast self-examination in detection of small cancers of the breast. *Cancer*, **54**, 1443.

SMITH, E. & BURNS, T. (1985). The effects of breast self-examination in a population based cancer registry: a report of differences in extent of disease. *Cancer*, **55**, 432.

UK TRIAL OF EARLY DETECTION OF BREAST CANCER GROUP (1981). Trial of early detection of breast cancer: description of method. *Br. J. Cancer*, **44**, 618.

VERBECK, A.L.M., HENDRICKS, J.H.L.L., HOLLAND, R. and 3 others (1984). Reduction in mortality through mass screening with modern mammography: first results of the Nijmegen project 1975–1981. *Lancet*, **i**, 1222.

WEISS, N.S. (1983). Control definition in case control studies of the efficacy of screening and diagnostic testing. *Am. J. Epidemiol.*, **118**, 457.